# The value of computed tomography for head trauma in patients presenting with out-of-hospital cardiac arrest before emergency percutaneous coronary intervention

**DOI:** 10.1007/s12471-023-01807-x

**Published:** 2023-08-24

**Authors:** Lena Bosch, Saskia Z. H. Rittersma, Bart H. van der Worp, Adriaan O. Kraaijeveld, George Vlachojannis, Pim van der Harst, Michiel Voskuil

**Affiliations:** 1 Department of Cardiology, University Medical Centre Utrecht, Utrecht, The Netherlands; 2 Department of Neurology, University Medical Centre Utrecht, Utrecht, The Netherlands

**Keywords:** Out-of-hospital cardiac arrest, ST-elevation myocardial infarction, Intracranial haemorrhage, Computed tomography, Head injury

## Abstract

**Introduction:**

Out-of-hospital cardiac arrest (OHCA) caused by an ST-elevation myocardial infarction (STEMI) is often accompanied by a sudden loss of consciousness that may cause the patient to collapse with resulting head trauma, leading to a suspicion of possible intracranial haemorrhage. To rule out intracranial haemorrhage before emergency percutaneous coronary intervention (PCI), emergency computed tomography (CT) of the head might be useful but also causes a delay in percutaneous STEMI treatment.

**Methods:**

The medical records of all adult patients that presented with OHCA to the emergency department (ED) of the University Medical Centre Utrecht (UMCU), the Netherlands between 16 February 2020 and 16 February 2022 were reviewed.

**Results:**

A total of 263 patients presented to the ED with an OHCA; 50 presented with a STEMI requiring emergency PCI. Thirty-nine (78%) patients with a STEMI were immediately referred to the catheterisation laboratory and 11 (22%) STEMI patients underwent a CT scan prior to emergency angiography; in no case was PCI deferred on the basis of the CT findings. The dominant indication for CT of the head was collapse, reported by 10 patients and resulting in a visible traumatic head injury in 7 patients. In none of the patients was intracranial haemorrhage detected. However, there was a delay between presentation to the ED and arrival at the catheterisation laboratory in patients who underwent CT of the head (mean 63 ± 25 min) before emergency PCI compared to patients without a CT scan (mean 37 ± 21 min).

**Conclusion:**

CT of the head did not result in a diagnosis of intracranial haemorrhage or deferral of PCI but did delay PCI treatment for STEMI in patients presenting with OHCA.

## What’s new?

- Performing computed tomography of the head before emergency angiography in ST-elevation myocardial infarction (STEMI) patients presenting with out-of-hospital cardiac arrest (OHCA) results in a significant delay in primary percutaneous coronary intervention.
- Intracranial haemorrhage is a rare complication after traumatic OHCA caused by STEMI.

## Introduction

An estimated 50% of out-of-hospital cardiac arrests (OHCAs) are due to a primary cardiac cause [1]. Cardiac ischaemia, resulting in ventricular arrhythmias, is frequently the underlying mechanism of cardiac arrest. A sudden loss of consciousness may lead to collapse, possibly causing traumatic (brain) injury. If an ST-elevation myocardial infarction (STEMI) is the underlying cause of the OHCA, emergency angiography is indicated, often followed by primary percutaneous coronary intervention (PCI). Administration of dual antiplatelet inhibitors and heparin is mandatory in the case of primary PCI, and is contra-indicated if intracranial haemorrhage is present. To rule out intracranial haemorrhage, emergency computed tomography (CT) of the head is often performed before emergency angiography in patients with visible head trauma. This decision may increase both door-to-balloon time and myocardial damage as well as worsening (cardiac) outcomes [2].

To gain insight into the necessity of ruling out intracranial haemorrhage before emergency angiography in patients presenting with OHCA and STEMI as well as a traumatic head injury, we retrospectively investigated all patients presenting to our centre with an OHCA.

## Methods

### Data collection

All adult patients (age ≥ 18 years) presenting with an OHCA to the emergency department (ED) of the University Medical Centre Utrecht (UMCU), the Netherlands, between 16 February 2020 and 16 February 2022 were included. The medical records were reviewed and medical data were manually extracted by the investigator. All analyses were performed on anonymised data. The evaluation and diagnosis made at the ED at the time of presentation were adopted, meaning no re-evaluation of patient data, such as electrocardiogram (ECG) interpretation, was performed. To determine the cause of death, the interpretation of the treating physician at the time of presentation was also adopted (even if no definite diagnosis was made or no autopsy performed). First troponin I and first creatine kinase-myocardial band (CK-MB) were only reported when blood was drawn at the ED. Left ventricular ejection fraction was categorised as normal (≥ 50%), reasonable (40–50%), moderate (30–39%) or poor (< 30%).

### Ethics review

This study does not fall under the scope of the Dutch Medical Research Involving Human Subjects Act (WMO). It therefore does not require approval by an accredited medical ethics committee in the Netherlands. However, an independent quality check was carried out at the UMCU to ensure compliance with legislation and regulations (regarding informed consent, data management, privacy aspects and legal aspects).

### Statistics

The normality of each continuous parameter was assessed using histograms, QQ plots and Kolmogorov-Smirnov tests. In the case of normal distribution we used a *t*-test, and when the data was not normally distributed a Mann-Whitney U test. For non-binary data Pearson’s chi-square test was used. Data are presented as median with interquartile range unless stated otherwise.

## Results

A total of 263 patients presented with an OHCA to the ED of the UMCU between 16 February 2022 and 16 February 2022 (Tab. 1). Seventy-seven percent (*n* = 203) of the patients were male, with a median age of 65 years (range 18–91 years). The initial rhythm was shockable (ventricular tachycardia or ventricular fibrillation) in 167 (63%) patients. Fifty-four percent (*n* = 143) of the resuscitations were for cardiac causes. A total of 175 (67%) patients died after OHCA, 93 (35%) thereof immediately at the ED and 82 (31%) during their hospital stay (mainly in the intensive care unit). Sixty-nine of 143 patients (48%) presenting with an OHCA of primary cardiac aetiology died.

**Table 1:** Characteristics of the 263 patients that presented to the emergency department (*ED*) with an out-of-hospital cardiac arrest (*OHCA*). Data are depicted as total number (percentage of total) or median (range). *PEA* pulseless electrical activity, *ICU* intensive care unit

	Total (*n* = 263)
*Sex*	
Male	203 (77%)
Female	60 (23%)
Age, years	65 (18–91)
*Initial rhythm*	
Shockable	167 (63%)
PEA/asystole	94 (36%)
*Cause of OHCA*	
Cardiac	143 (54%)
Non-cardiac	95 (36%)
Unknown	25 (10%)
Emergency angiography	35 (13%)
*Mortality*	
Total	175 (67%)
At the ED	93 (35%)
In the ICU	82 (31%)

### Emergency angiography

**Fig. 1:**
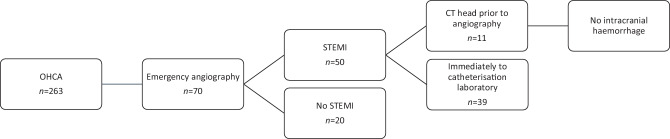
A total of 263 patients presented to the emergency department with an out-of-hospital cardiac arrest (*OHCA*). Seventy patients were immediately referred to the catheterisation laboratory for emergency angiography. The primary reason for emergency angiography was an ST-elevation myocardial infarction (*STEMI*) in 50 patients. Prior to catheterisation laboratory presentation computed tomography (*CT*) of the head was performed in 11 patients that presented with a STEMI. No intracranial haemorrhage was detected

In total, 70 patients (27%) were immediately referred from the ED to the catheterisation laboratory (Fig. 1). The primary reason for emergency angiography was ECG findings consistent with a STEMI in 50 patients. The other 20 patients without a STEMI were referred to the catheterisation laboratory for various reasons, such as initiation of extracorporeal life support, diffuse ischaemia on the ECG suspicious of a left main coronary artery stenosis, or in the exceptional case of percutaneous intervention for a left ventricular assist device outflow tract obstruction. These cases are beyond the scope of this study and were excluded from further analysis.

### CT scan of the head

Thirty-nine (78%) patients with suspicion of a STEMI were immediately referred to the catheterisation laboratory and 11 (22%) STEMI patients underwent CT of the head prior to emergency angiography. Intracranial haemorrhage was not detected on any of the CT scans performed and in no patient was emergency angiography deferred on the basis of the CT findings. The dominant indication for CT of the head was a possible traumatic collapse, reported in 10 of 11 patients (91%) that presented with a STEMI. In 7 patients (64%) this resulted in a visible traumatic head injury, ranging from small skin lacerations to multiple facial fractures. Six of the 20 patients (30%) without a STEMI referred for emergency angiography first underwent CT of the head; in this group no intracranial haemorrhage was diagnosed. In addition to the CT scans performed prior to angiography, in 4 patients (8%) presenting with a STEMI and collapse CT of the head was performed immediately after emergency angiography. None of these patients were diagnosed with intracranial haemorrhage.

### Comparison of STEMI patients with and without head CT scan

**Fig. 2:**
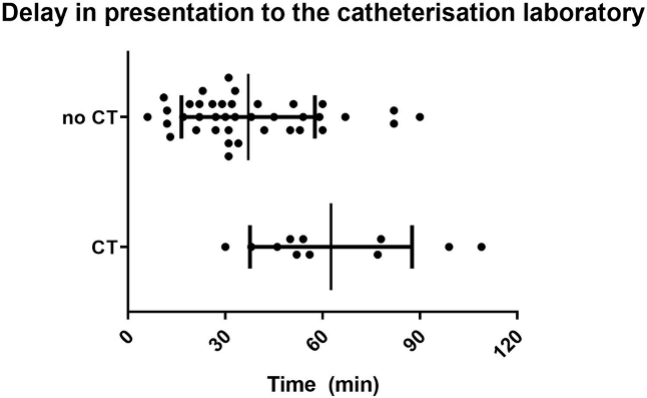
Delay between presentation to the emergency department and arrival at the catheterisation laboratory in patients who first underwent computed tomography (*CT*) of the head (*n* = 11, mean 63 ± 25 min) before emergency percutaneous coronary intervention compared to patients in whom head CT was not performed (*n* = 39, mean 37 ± 21 min, *p* = 0.002)

Table 2 summarises the characteristics of patients with a suspected STEMI that did (*n* = 11) and did not (*n* = 39) undergo a head CT scan prior to angiography. No significant differences were detected. The patients presenting with a STEMI that underwent CT before primary PCI had an average time from arrival at the ED to arrival at the catheterisation laboratory of 63 ± 25 min compared to 37 ± 21 min (*p* = 0.002) in those who went directly to the catheterisation laboratory (Fig. 2).

**Table 2:** Comparison of ST-elevation myocardial infarction patients who did or did not undergo computed tomography (*CT*) of the head prior to angiography. Continuous variables are presented as median with interquartile range except for age, which is presented as mean ± SD. The group variables are presented as total number (percentage)

	CT (*n* = 11)	No CT (*n* = 39)	*p*
Age	68 ± 12	59 ± 15	0.40
*Initial rhythm*			0.34
Shockable	11 (100%)	36 (92%)	
PEA/asystole	0 (0%)	3 (8%)	
*DAPT timing*			0.89
Ambulance	1 (9%)	14 (36%)	
Catheterisation laboratory	8 (73%)	25 (64%)	
No DAPT	2 (18%)	0	
*Culprit vessel*			0.31
LAD	3 (27%)	14 (36%)	
LCx	1 (9%)	8 (21%)	
RCA	3 (27%)	3 (8%)	
No culprit vessel	4 (36%)	14 (36%)	
pH (ED)	7.26 (7.20–7.33) (*n* = 10)	7.22 (7.09–7.27) (*n* = 34)	0.14
First troponin I (ED) (ng/l)	403 (169–769) (*n* = 9)	457 (129–1198) (*n* = 37)	0.96
First CK-MB (ED) (µg/l)	6.6 (4.6–14.7) (*n* = 9)	6.5 (4.6–13.0) (*n* = 37)	0.89
CK-MB max (µg/l)	146 (68–288) (*n* = 10)	177 (59–379) (*n* = 29)	0.09
CK max (U/l)	1424 (1087–1815) (*n* = 9)	2422 (942–5037) (*n* = 28)	0.97
*LV function (2* *±* *1 days after OHCA)*	*n* =8	*n* = 22	0.05
Normal	0 (0%)	3 (14%)	
Reasonable	7 (88%)	7 (32%)	
Moderate	0 (0%)	7 (32%)	
Poor	2 (23%)	5 (23%)	
*LV function (4* *±* *3 months after OHCA)*	*n* =6	*n* = 11	0.08
Normal	1 (17%)	7 (64%)	
Reasonable	4 (67%)	1 (9%)	
Moderate	1 (17%)	2 (18%)	
Poor	0 (0%)	1 (9%)	
Mortality	4 (36%)	16 (41%)	0.78

*PEA* pulseless electrical activity, *DAPT* dual antiplatelet therapy, *LAD* left anterior descending coronary artery, *LCx* left circumflex artery, *RCA* right coronary artery, *ED* emergency department, *CK-MB* creatine kinase-myocardial band, *CK* creatine kinase, *LV* left ventricular, *OHCA* out-of-hospital cardiac arrest

## Discussion

In our cohort, intracranial haemorrhage was not detected in patients presenting with OHCA and STEMI, even in the presence of a visible external head injury. According to current guidelines, a primary PCI strategy is recommended in patients resuscitated from cardiac arrest and ECG findings consistent with STEMI. This is followed by CT of the head and/or CT pulmonary angiography if coronary angiography fails to identify causative lesions [3, 4].

The added value of and indications for a head CT scan before emergency angiography in OHCA patients have not been established. A meta-analysis by Petek and colleagues studied the diagnostic yield of CT scanning in non-traumatic OHCA patients in general, not specifically for cardiac causes. Between 0.3% and 24% of head CT scans led to a diagnosis such as subarachnoid or meningeal haemorrhage that was not necessarily the cause of the OHCA [5]. One study performed in a single centre in San Francisco prospectively investigated the value of head CT in all OHCA patients arriving at their ED with return of spontaneous circulation (ROSC), including patients presenting with STEMI [6]. Their goal was to identify the incidence of intracranial haemorrhage as a cause of OHCA. A total of 85 CT scans were performed in 95 patients who had sustained ROSC after OHCA. In 3 patients intracranial haemorrhage was detected, 2 of whom presented with OHCA based on a non-shockable rhythm. No information was available about the type and cause of intracranial haemorrhage and whether collapse or trauma was present. In 10 patients CT of the head was deferred, in 6 of these because of an emergency PCI.

In the majority (73%) of patients in our cohort that underwent CT, antithrombotic treatment was delayed until after the scan had been performed. When an emergency CT scan was performed, PCI was delayed on average by 26 min. In this small retrospective study the delay of 26 min did not lead to demonstrable cardiac damage (measured by peak CK and CK-MB) or increased mortality. However, it is known that infarct size and mortality rates increase as door-to-balloon time increases [2, 7]. Shortening door-to-balloon time reduces 1‑year mortality and every reduction of door-to-balloon time by 30 min resulted in a reduction of 1‑year mortality [8, 9]. Therefore efforts to shorten door-to-balloon time should apply for all patients. The risk of missing a possible haemorrhage should be taken into account. Based on our retrospective study we cannot conclude that prior CT scanning in this clinical scenario should not be performed, but our findings suggest that even in the case of serious traumatic injury after OHCA, the risk of intracranial haemorrhage is low. Therefore, we believe that in the majority of cases deferring head CT and immediately proceeding to emergency PCI might be reasonable.

Our study has several limitations. Follow-up data were not available for all patients due to transfers to other hospitals. Even though we detected no intracranial haemorrhage, the patient numbers of the current cohort are low and therefore our conclusions are hypothesis generating. The possible consequences of missing an intracranial haemorrhage prior to emergency PCI may be clinically relevant as treatment with dual antiplatelet therapy and heparin could have detrimental effects.

Finally, in every hospital logistics and the amount of time required to perform a CT scan can differ. A CT scanner located nearby/at the ED will probably reduce the delay in performing a CT scan, thereby facilitating rapid proceeding to PCI.

In conclusion, in this retrospective study CT of the head did not result in a diagnosis of intracranial haemorrhage or deferral of PCI but did delay PCI treatment for STEMI in patients presenting with OHCA.
